# Multidimensional analysis of honey from Eastern Anatolia (Kars): Pollen spectrum, physicochemical properties, and antimicrobial activity

**DOI:** 10.1371/journal.pone.0327861

**Published:** 2025-07-09

**Authors:** Salih Akpınar, Neslihan Mutlu

**Affiliations:** Department of Biology, Faculty of Science and Letters, Kafkas University, Kars, Türkiye; Universidad San Francisco de Quito - Campus Cumbaya: Universidad San Francisco de Quito, ECUADOR

## Abstract

This study investigates the physicochemical properties, pollen composition, and antimicrobial activity of honey samples collected from Kars province, Türkiye. A total of 30 honey samples were analyzed for moisture content (13.9–18.0%), pH (3.44–3.92), electrical conductivity (0.138–0.343 mS/cm), free acidity (13.9–31.5 meq/kg), diastase activity (4.93–28.1 DN), hydroxymethylfurfural (HMF) levels (3.5–37.1 mg/kg), sugar profile (fructose: 32.8–41.0%, glucose: 26.2–32.6%, sucrose: 0.1–2.7%, maltose: 1.4–2.7%), and proline content (288.87–1272.9 mg/kg). Pollen analysis identified 52 taxa from 30 botanical families, with Fabaceae, Boraginaceae, Lamiaceae, Asteraceae, and Brassicaceae being the most dominant. Of the honey samples, 27 were classified as multifloral and 3 as unifloral. Total pollen counts per 10 g (TPC-10 g) of honey ranged between Group II and Group V categories (2000 to > 100000 grains). The antimicrobial activity of the honey was evaluated against *Escherichia coli*, *Klebsiella pneumoniae*, *Pseudomonas aeruginosa*, *Staphylococcus aureus*, *Enterococcus faecalis*, and *Bacillus subtilis* using the agar well diffusion method. Inhibition zones ranged from 11 ± 0.55 mm to 24 ± 1.2 mm, with greater efficacy observed against Gram-positive bacteria. Significant correlations were found between diastase activity and *Staphylococcus aureus* (r = 0.704, p < 0.01) and *Enterococcus faecalis* (r = 0.518, p < 0.01), as well as between proline content and *Klebsiella pneumoniae* (r = 0.454, p < 0.05), *Pseudomonas aeruginosa* (r = 0.384, p < 0.05), *Staphylococcus aureus* (r = 0.439, p < 0.05), and *Bacillus subtilis* (r = 0.435, p < 0.05). The sugar composition complied with international standards, with fructose and glucose being the predominant sugars. HMF levels remained within acceptable limits, suggesting appropriate storage and processing conditions. Overall, the findings highlight the high quality and antimicrobial potential of Kars honey, supporting its value as a natural and functional food product.

## Introduction

Honey is of great importance due to its nutritional, medicinal, and economic value. From a nutritional perspective, it is a natural sweetener rich in carbohydrates, primarily fructose and glucose, providing a rapid source of energy [1]. Additionally, honey contains various vitamins, minerals, and antioxidants that contribute to its health benefits [2]. The antioxidant properties of honey, attributed to its phenolic compounds, play a crucial role in combating oxidative stress and may help prevent chronic diseases [3].

Medically, honey is recognized for its antibacterial and anti-inflammatory properties, making it effective in wound healing and a natural remedy for various ailments [4]. Its ability to inhibit the growth of antibiotic-resistant bacteria further highlights its therapeutic potential [5]. In addition to its broad-spectrum antibacterial effects, recent studies have also demonstrated that honey exhibits antibiofilm activity against both susceptible and multidrug-resistant pathogens, thereby enhancing its biomedical relevance [6,7]. Furthermore, its role in traditional medicine across cultures underscores its long-standing significance in healthcare [8]. Honey has also been associated with improved gastrointestinal health by promoting beneficial gut microbiota, which can enhance nutrient absorption and overall digestive well-being [9]. Its therapeutic applications extend to the management of chronic diseases, including diabetes and cardiovascular conditions, due to its ability to modulate blood sugar levels and exert anti-inflammatory effects [10]. Moreover, the increasing use of honey as a natural sweetener in functional foods reflects a growing shift toward healthier dietary choices among consumers [11].

Various physical and chemical parameters are assessed to determine honey quality. Moisture content is one of the most critical factors, as lower moisture levels reduce the risk of fermentation and ensure a longer shelf life [12]. Additionally, pH and acidity provide insights into honey’s freshness and microbial resistance, with pH values generally ranging between 3.7 and 4.5 [13]. Another important quality indicator is HMF content, a compound formed during the aging process; elevated levels may indicate heat treatment or adulteration [14]. Furthermore, diastase activity serves as a marker for honey’s natural and unprocessed state, with higher diastase levels suggesting freshness [15].

Pollen analysis plays a crucial role in determining the botanical origin and, consequently, the quality of honey. This method involves identifying and quantifying pollen grains in honey samples, which serve as natural markers of the plants visited by bees during nectar collection [1,16]. Studies have shown that the pollen profile significantly influences honey’s quality and authenticity, distinguishing between monofloral and polyfloral characteristics based on specific pollen types [17]. Additionally, melissopalynological analysis has been employed to evaluate the impact of environmental factors on the pollen spectrum of honey, revealing how agricultural practices can alter its floral composition [18]. Overall, pollen analysis not only aids in quality control but also enhances our understanding of bee foraging behavior and local biodiversity [19]. In countries with rich plant diversity, such as Türkiye, these analyses are essential for highlighting the authenticity and regional identity of honey. A comprehensive evaluation of all these parameters is necessary for an accurate assessment of honey quality.

This study aims to determine the characteristic properties of honey samples from Kars province by analyzing key chemical and physical parameters, including their pollen composition, total pollen count, pH, sugar content, moisture level, proline, and HMF levels. Additionally, the study seeks to provide a comprehensive scientific evaluation of the chemical and biological properties of Kars honey by assessing its antimicrobial activity.

## Materials and methods

### Honey samples

Kars province is located in the Erzurum-Kars section of the Eastern Anatolia region, between 42°10′ and 44°49′ east longitudes and 39°22′ and 41°37′ north latitudes. With a surface area of 10127 km², Kars has an average altitude of 1768 meters. The province has a continental climate, making it one of the coldest regions in Türkiye. Summers are short and mild, whereas winters are long and characterized by severe cold. The region records an annual average of 120 days of snowfall, while frost events persist for approximately 180 days [20].

A total of 30 honey samples were collected in August and September 2023 from different districts of Kars province, including Sarıkamış, Arpaçay-Akyaka, and Kağızman (six samples each), Kars city center (seven samples), and Susuz (five samples). Specifically, samples 1–3 were obtained from Arpaçay, 4–6 from Akyaka, 7–12 from Kağızman, 13–19 from the city center, 20–25 from Sarıkamış, and 26–30 from Susuz. At least 500 grams of honey were collected from each beehive. The jars were labeled with the collection location, date, and producer name before being transported to the laboratory for analysis. All honey samples were stored at room temperature in a dry and dark environment throughout the research period. All honey samples were obtained from local producers and commercial vendors. No specific permits were required for sample collection, as the study did not involve fieldwork or access to protected areas.

### Pollen analysis

Pollen analysis of the honey samples was conducted following standard methods recommended by international beekeeping institutions [21–23]. For sample preparation, 10 grams of homogenized honey were dissolved in 20 ml of distilled water in a water bath at 40–45°C for 10–15 minutes. The solution was centrifuged at 3500–4000 rpm for 45 minutes, and the supernatant was carefully removed. The remaining residue was dried and mixed with a glycerin-gelatin medium containing basic fuchsin dye. This mixture was transferred onto microscope slides, covered with a coverslip, and left to dry for approximately 12 hours. Microscopic examination was performed using an Olympus CX21 microscope at 40X magnification. At least 200 pollen grains were counted per sample, and their types were identified using reference collections and relevant literature. Pollen grains were categorized into four frequency classes: dominant (≥ 45%), secondary (16–44%), minor (3–15%), and trace (< 3%) [21]. This classification was applied to determine the botanical origin and floral diversity of the honey samples.

### Determination of TPC-10 g in honey

The TPC-10 g in honey was determined using the method described by Moar (1985) [23]. A homogenized stock honey sample was prepared, and 10 g was mixed with 20 ml of distilled water. A tablet containing 17197 *Lycopodium* spores was added to the mixture. The tubes were incubated in a water bath at approximately 45°C for 10–15 minutes to dissolve the tablet, with occasional stirring using a glass rod. After complete dissolution, basic fuchsin was added to stain the pollen and spores. The mixture was centrifuged at 3500–4000 rpm for 45 minutes, and the supernatant was discarded. The sediment was mixed with 0.1 ml of 50% glycerin and homogenized.

From this mixture, 0.01 ml was transferred into a second tube containing 0.09 ml of glycerin and homogenized again. A 0.01 ml aliquot of the second mixture was placed onto a microscope slide, covered with an 18 × 18 mm coverslip, and prepared for microscopic examination. Two tubes were prepared for each honey sample, and one slide was prepared from each tube. The counts from the two slides were averaged, and the TPC-10 g was calculated using the following formula: TPC = (Pollen count × 17197)*/Lycopodium* spores counted.

Honey samples were classified based on their TPC-10 g values into five categories: very low pollen honey (< 2000), normal pollen honey (2000–10000), high pollen honey (10000–50000), very high pollen honey (50000–100000), and mega-rich pollen honey (> 100000) [24,25].

### Investigation of antimicrobial properties of honey

The antimicrobial properties of honey were evaluated using six bacterial strains: *Escherichia coli* ATCC 25922, *Staphylococcus aureus* ATCC 25923, *Klebsiella pneumoniae* ATCC 700603, *Bacillus subtilis* ATCC 6051, *Pseudomonas aeruginosa* ATCC 27853, and *Enterococcus faecalis* ATCC 29219. The agar well diffusion method was used to evaluate the antimicrobial activity of the honey samples. Bacterial cultures were grown in Tryptic Soy Broth (Difco, 30 g/L) and standardized to a 0.5 McFarland turbidity standard to ensure uniform bacterial counts. Sterile Mueller Hinton Agar was prepared, cooled to a safe handling temperature, and mixed with 100 μl of bacterial suspension. This mixture was poured into sterile Petri dishes and allowed to solidify. After solidification, four wells of equal size were made in each Petri dish using a sterile cork borer. Honey dilutions at concentrations of 25%, 50%, and 75% were prepared, and 100 μl of each dilution was added to the wells. Plates were incubated at 37°C for 24 hours. After the incubation period, inhibition zones around the wells were measured in millimeters [26,27].

### Determination of insoluble matter

Insoluble matter content was determined according to the harmonized methods of the European Honey Commission [28]. Approximately 20 g of homogenized honey was dissolved in warm distilled water and filtered through a pre-weighed sintered glass crucible (porosity no. 2). The residue was thoroughly rinsed with hot distilled water to remove any remaining sugars and soluble materials. The crucible was then dried in a laboratory oven (Selecta Digitheat) at 135 °C until constant weight was achieved. After drying, it was cooled in a desiccator and weighed using an analytical balance (Shimadzu ATX 224) with a sensitivity of 0.1 mg. The difference in mass was used to calculate the amount of insoluble matter, and the results were expressed as g/100 g of honey.

### Diastase activity

Diastase activity was measured according to the Phadebas method described by the International Honey Commission (IHC) [29]. Honey samples (1 g) were dissolved in acetate buffer (pH 5.2) and incubated at 40 °C with Phadebas tablets (Magle Life Sciences, Sweden) for 60 minutes. The reaction was terminated with NaOH solution, and absorbance was read at 620 nm using a Shimadzu UV-1800 spectrophotometer. Results were expressed in Schade units, and each sample was analyzed in triplicate.

### Electrical conductivity

Electrical conductivity was determined using a Hach HQ40D portable multi-parameter meter (USA), equipped with an Intellical CDC401 conductivity probe containing a platinum cell. A 20% (w/v) honey solution was prepared in deionized water. Measurements were carried out at 20 °C, and the results were expressed in millisiemens per centimeter (mS/cm) [28]. Each sample was measured in triplicate.

### Free acidity

Free acidity was measured by titration with 0.1 M NaOH followingIHC protocols. Ten grams of honey were dissolved in 75 mL of carbon dioxide-free distilled water. The solution was stirred and titrated with 0.1 M sodium hydroxide until the pH reached 8.30, monitored using a calibrated pH meter (Hanna Instruments HI2211, Romania). Results were expressed as milliequivalents of acid per kilogram of honey (meq/kg), and all measurements were performed in triplicate [28].

### HMF content

HMF content was analyzed using a Shimadzu LC-20AT high-performance liquid chromatography (HPLC) system equipped with a SPD-20A UV detector, set to 285 nm. The separation was performed using a Phenomenex Luna C18 column (250 mm × 4.6 mm, 5 µm) at room temperature. The mobile phase consisted of water:methanol (90:10, v/v), and the flow rate was 1.0 mL/min. Samples were filtered through a 0.45 µm syringe filter prior to injection. A 20 µL injection volume was used, and results were expressed in mg/kg [30].

### Moisture content

Moisture content was determined using the refractometric method recommended by the IHC [28]. Prior to measurement, honey samples were gently heated in a water bath at 50 °C for 10–15 minutes to dissolve any sugar crystals. After homogenization, a drop of each sample was placed onto the prism of an Abbé refractometer (Atago NAR-1T, Japan) at 20 °C. Refractive index values were recorded and converted into moisture content (%) using Wedmore’s table. Each sample was analyzed in duplicate, and the average value was reported.

### pH analysis

The pH of honey samples was measured using a 10% (w/v) honey solution prepared in CO₂-free distilled water. The pH values were recorded using a Hanna Instruments HI2211 digital pH meter calibrated with standard buffer solutions (pH 4.01, 7.00, and 9.21) before each measurement. All analyses were conducted at room temperature (20 ± 2 °C) and repeated twice for each sample [28].

### Proline content

Proline content was determined according to the IHC protocol [28]. One gram of honey was dissolved in distilled water, and the sample was reacted with acidic ninhydrin solution. The reaction mixture was incubated at 100 °C for 15 minutes. After cooling, absorbance was measured at 510 nm using a UV-Vis spectrophotometer (Shimadzu UV-1800, Japan). Results were calculated using a calibration curve prepared with L-proline standard solutions and reported in mg/kg [31].

### Sugar composition

Sugar analysis was performed using a Shimadzu LC-20AT HPLC system coupled with a pulsed amperometric detector and a C18 column (250 mm × 4.6 mm, 5 µm). Honey samples were diluted in ultrapure water, filtered through 0.45 µm membrane filters, and injected into the HPLC system. The mobile phase was acetonitrile:water (75:25, v/v), with a flow rate of 1.0 mL/min. Calibration curves were prepared using standard solutions of fructose, glucose, and sucrose. Results were expressed as percentages of each sugar relative to total sugar content [32].

### Statistical analysis

All tests were performed in triplicate. Data analysis was performed using IBM SPSS Statistics 20 version. The statistical significance (p < 0.05) of the sample composition values was tested by means of one-way analysis of variance (ANOVA) using Tukey’s post hoc comparison test. Detailed results of all physicochemical parameters and their statistical comparisons are provided in S1 Table (see Supporting Information). Finally, the relationship between insoluble matter content, diastase activity, electrical conductivity, free acidity, HMF, moisture content, pH, proline, sugar profile, and antibacterial activity evaluated in honey was determined by Spearman’s correlation test for a sample size of n = 30.

## Results and discussion

### Pollen analysis

**Fig 1:**
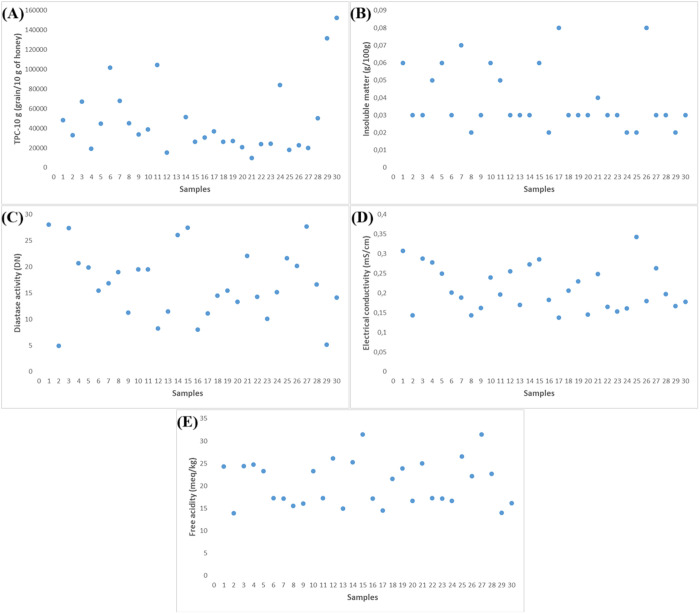
Physicochemical properties of honey samples: (A) TPC-10 g (grain/10 g of honey), (B) Insoluble matter (g/100g), (C) Diastase activity (DN), (D) Electrical conductivity (mS/cm), and (E) Free acidity (meq/kg).

Pollen analysis in honey is a crucial tool for determining its botanical origin of honey [33,34]. Studies on pollen diversity in honey help determine whether the honey is unifloral or multifloral [33,35]. Pollen analysis results are given in Table 1. In this study, a total of 52 taxa belonging to 30 families were identified in 30 honey samples. The number of plant taxa in the analyzed honey samples ranged from 13 to 39. The dominant families were Fabaceae (*Astragalus*, *Onobrychis*, *Trifolium*, *Vicia*), Boraginaceae (*Myosotis*, *Echium*), Lamiaceae (*Salvia*), Asteraceae, and Brassicaceae. The most frequently observed families in the honey samples included Fabaceae (n = 30), Lamiaceae (n = 30), Boraginaceae (n = 29), Asteraceae (n = 29), Brassicaceae (n = 28), Rosaceae (n = 28), and Apiaceae (n = 28). Among the identified taxa, 21 were present in more than half of the honey samples, with *Astragalus* (n = 30), *Onobrychis* (n = 30), *Salvia* (n = 30), *Vicia* (n = 30), *Echium* (n = 28), *Trifolium* (n = 28), *Eryngium* (n = 27), and *Mentha* (n = 26) being the most common. Of the 30 honey samples, 27 were classified as multifloral, while 3 were identified as unifloral, represented by *Myosotis* pollen. The pollen analysis results showed significant similarity to those of a 2018 study conducted in the Kars region of Türkiye and to the pollen composition of Ardahan honey samples [36,37]. These findings are largely consistent with previous melissopalynological studies conducted in northeastern Türkiye. For instance, Gençay Çelemli et al. (2018) reported that Fabaceae, Boraginaceae, and Asteraceae were among the most frequently encountered families in 100 honey samples collected from Kars [36]. Onobrychis radiata, Trifolium nigrescens, and Echium vulgaris were dominant or secondary in the majority of the samples, aligning closely with the taxa observed in the present study. Similarly, Şık et al. (2017) found Astragalus spp., Apiaceae, Brassicaceae, and Fabaceae to be common in Ardahan honeys, all of which were also present in our samples [37]. The total number of identified taxa and families in our study (52 taxa from 30 families) is comparable to the floristic richness reported by Gençay Çelemli et al. (2018), who recorded 54 taxa from 30 families [36]. Moreover, the high occurrence of Astragalus, Onobrychis, Salvia, and Echium confirms the dominance of local steppe and meadow flora, especially in high-altitude continental climates. These similarities indicate that honey produced in the Kars region reflects a shared regional flora with neighboring provinces such as Ardahan, further validating the floral origin and geographical distinctiveness of the studied samples. Based on the TPC-10 g of honey, one sample was classified in Group II, 19 samples in Group III, and five samples each in Groups IV and V. No honey sample was found to contain very low pollen concentrations (Fig 1).

**Table 1:** Botanical origin of the honey samples.

Family	Taxa	1	2	3	4	5	6	7	8	9	10	11	12	13	14	15	16	17	18	19	20	21	22	23	24	25	26	27	28	29	30
**Amaranthaceae**	**Amaranthaceae**		2		0.5		3.5	0.5	2.5	0.5	1	7			0.5	1													2.5		
**Amaryllidaceae**	*Allium*	1.5		0.5	5.5	5.5	6	6.5	7.5	7	1	5.5	1	4	1		1.5	0.5	1.5	1		1			0.5			0.5			
	**Amaryllidaceae**			1	2	1	0.5	1	2	0.5		2	0.5			0.5	1					1.5							1		
**Apiaceae**	**Apiaceae**		0.5	1.5		0.5	1.5	0.5	1	0.5	1.5	1.5		0.5	1						0.5				1						
	*Eryngium*	3.5	2	1	0.5	4	3	1	2	1	0.5	3	5.5	2	8	9	4	1	1	2.5	2.5	3	0.5			2.5		1.5	1.5	2	2
**Asteraceae**	*Achillea*		2.5	11	11.5	0.5	4	1.5	2	0.5	2.5	3				0.5	0.5		0.5		4	8	1.5		1	2	1	0.5			5.5
	*Anthemis*	3	5	5	5	0.5	4	1	1.5	0.5	1.5	2.5			0.5	0.5	0.5				2.5	4.5	1		0.5	1.5			1		1.5
	*Artemisia*			1	1		0.5								1		0.5	0.5	0.5				0.5			0.5	0.5				1
	*Carduus*	0.5	0.5	2				4	1.5	0.5	0.5	0.5		0.5		0.5	1		1			1							1		1.5
	*Carthamus*				0.5		0.5	0.5							0.5																
	*Centaurea*	0.5	1.5	2	2.5			0.5	1	0.5	0.5	1.5	4.5	1	1	0.5	6	2	1.5		4	0.5	1.5						1	4	4
	*Cichorium*	5		3	5.5	0.5	1				0.5	0.5				1			0.5	1.5		3.5									
	*Cirsium*				3.5					0.5	1			1		0.5					1										
	*Helianthus*	0.5		0.5					0.5													1									0.5
	*Hieracium*	0.5			5.5	0.5	0.5													0.5											
	*Onopordum*	1.5	0.5				0.5			0.5	2			0.5	0.5	0.5			0.5			9.5	2		0.5		1				2
	*Senecio*	0.5								0.5	0.5				0.5	0.5						0.5									
	*Sonchus*									0.5	0.5	0.5										0.5			0.5				1.5		
	*Taraxacum*	2	1	12	8.5	1	2.5				1	3		2.5	1	0.5			1	1.5		2.5									
**Boraginaceae**	*Anchusa*			0.5		1.5			0.5	1		0.5				0.5									0.5						
	*Echium*	4	19.5	3	0.5	4.5	4	4	4.5	3.5	2.5	2	15.5	12	2	12.5	19.5	6	3.5	3.5	8.5	2		13	14	2.5	8	19.5	24.5		13
	*Myosotis*	0.5	2	0.5			0.5			0.5								30	55	38			59	12	0.5	61.5					
	*Symphytum*			3.5		0.5		3		0.5			3	0.5	3	3	1.5			1.5		2.5		8	0.5		2.5	1	2		0.5
**Brassicaceae**	**Brassicaceae**	14.5	6.5	0.5	6	5	6	6	3	14	6	22	8.5	6.5	18	19.5	8	13.5	7.5	6	2.5	4.5	1.5			2.5	9	10.5	7	12	8
**Caprifoliaceae**	*Dipsacus*			1		1		0.5	0.5		1.5					0.5					0.5										
	*Scabiosa*		7.5					6	1		2.5	1				0.5			0.5		1.5		1	3.5			3				
**Caryophyllaceae**	**Caryophyllaceae**		0.5				4.5	0.5	1	0.5	3.5	0.5	9	2	20.5	6	6	1.5	2	7	2.5			3.5		2	5.5	6.5	6	10.5	6
**Cistaceae**	**Cistaceae**	1	0.5			2.5		1	1	12.5	3.5	0.5									1	2		2.5	4	1	2.5				3.5
**Cyperaceae**	**Cyperaceae**		0.5		0.5				0.5	0.5																					
**Euphorbiaceae**	*Euphorbia*				0.5	1	0.5	0.5		1	2	0.5						1		0.5		0.5									
**Fabaceae**	*Astragalus*	12	11.5	8.5	6.5	32	13.5	12	23.5	9.5	23	8	18.5	12	9.5	7	19	10	9.5	13	18	13.5	13	19.5	36	8.5	16.5	14	7	9.5	15
	*Lotus*	0.5	0.5	2.5	2	0.5		0.5	0.5	0.5	0.5	1	0.5		0.5	0.5			0.5		0.5	1		0.5	0.5		1		0.5		0.5
	*Onobrychis*	20.5	9	1.5	1	11	7.5	15	13	10.5	9.5	11	19	32.5	19	8.5	12.5	9.5	4.5	8.5	7.5	3	8	6.5	8	2.5	6.5	10.5	6.5	41	12.5
	*Trifolium*	6.5	9.5	9	2.5	3	6.5	4	3.5	2	7.5	6.5	0.5	1	0.5	2.5	3	3		3.5	4.5	3.5		4.5	7.5		4.5	4	4	7	4.5
	*Vicia*	3.5	1.5	4.5	3	7.5	2.5	1.5	2	5	3.5	2	4	2	0.5	4	5.5	6.5	0.5	3	6.5	2.5	0.5	6.5	5.5	4	5	8	10.5	1	3
**Juglandaceae**	*Juglans*								0.5		0.5				1	2			0.5												
**Lamiaceae**	**Lamiaceae**		2	3.5	6.5	1.5	4	4.5	3	4.5	2	1	2.5	2.5	2	1	3	3.5	3	1.5	4	7	3	2.5	5.5	2	13.5	4	5.5	5.5	1.5
	*Mentha*		1	0.5	2	0.5	1	1	0.5	0.5	0.5		3	1		0.5	0.5	2.5	0.5	1	2	2	1	3.5	2		4.5	5.5	7	2	4
	*Salvia*	4	6	5.5	11	3.5	7.5	11	8	6.5	4.5	2.5	2	10	4.5	4	2.5	0.5	1.5	3	8.5	5	4	5.5	3.5	1.5	7.5	8.5	4.5	2.5	6.5
**Liliaceae**	**Liliaceae**	0.5		0.5	0.5																					0.5				0.5	
**Malvaceae**	*Alcea*										0.5								0.5			1.5			3.5		2	1	2.5		
	*Tilia*			0.5	0.5			1		0.5											1.5										
**Moraceae**	*Morus*					0.5										0.5															
**Papaveraceae**	*Roemeria*		2	1.5		0.5				0.5																					
**Plantaginaceae**	*Plantago*			0.5	0.5						3.5	0.5			1		0.5					1									
**Poaceae**	**Poaceae**	1.5	0.5	0.5	0.5	2.5	0.5	2.5		0.5	1	0.5	1			3.5		1		1	3	3.5		2							
**Polygonaceae**	*Polygonum*	0.5		1.5				1	1.5	1.5	1	1				0.5		0.5		0.5	1	2		2.5	1	0.5		0.5			0.5
	*Rumex*			0.5	0.5						0.5					0.5				0.5	0.5	0.5									
**Ranunculaceae**	**Ranunculaceae**		0.5	0.5	0.5	0.5	3				0.5	1																			
**Rosaceae**	**Rosaceae**		2.5	2.5		1.5	10	7	8	6.5	3	6.5	1.5	5	2.5	5	2.5	7	2.5	0.5	9	3.5	1.5	4	2.5	4	4	4	2.5	2.5	2.5
**Solanaceae**	*Solanum*	6.5	1	6.5	3	3.5	0.5	0.5	2.5	4	2	1		1		1.5	1			0.5	2.5	2			1	0.5	2		0.5		0.5
**Urticaceae**	*Urtica*	5				1.5					0.5					0.5							0.5								

### Antimicrobial activity

The antimicrobial activity of honey samples against Gram-negative bacteria *Escherichia coli*, *Klebsiella pneumoniae*, *Pseudomonas aeruginosa* and Gram-positive bacteria *Enterococcus faecalis*, *Staphylococcus aureus*, and *Bacillus subtilis* was determined using the well diffusion method, and the results are presented in Table 2. Except for the 30th honey sample, all samples exhibited activity against at least one bacterium. The antimicrobial activity of honey samples from the Kars region varied depending on the bacterial species, with inhibition zones ranging from a minimum of 11 ± 0.55 mm to a maximum of 24 ± 1.2 mm. The inhibition zones against *E. coli* ranged from 12 ± 0.6 mm to 18 ± 0.9 mm, while for *K. pneumoniae*, the values varied between 11 ± 0.55 mm and 20 ± 1 mm. The inhibition zones for *S. aureus* ranged from a minimum of 12 ± 0.6 mm to a maximum of 18 ± 0.9 mm.

**Table 2:** Antibacterial activity (inhibition zone diameters in mm) of honey samples against tested bacterial strains at 25%, 50%, and 75% concentrations.

	*Escherichia coli*			*Klebsiella pneumoniae*			*Pseudomonas aeruginosa*			*Enterococcus faecalis*			*Staphylococcus aureus*			*Bacillus subtilis*		
Samples	75%	50%	25%	75%	50%	25%	75%	50%	25%	75%	50%	25%	75%	50%	25%	75%	50%	25%
1	15 ± 0.75^cde^	15 ± 0.75^bc^		15 ± 0.75 cd	12 ± 0.6^c^		13 ± 0.65^def^						17.5 ± 0.88^ab^	13 ± 0.65^bcd^	11 ± 0.55^c^			
2	14 ± 0.70^def^	11 ± 0.55^e^		12 ± 0.6^ef^	12 ± 0.6^c^													
3	16 ± 0.80^abcd^	16 ± 0.8^ab^		16 ± 0.8^bcd^	14 ± 0.7^abc^					14 ± 0.7^h^	12 ± 0.6^f^	16 ± 0.8^ab^	14 ± 0.7^def^	12 ± 0.6 cd		16 ± 0.8^cde^		
4	16 ± 0.80^abcd^	15 ± 0.75^bc^	13 ± 0.65^a^	16 ± 0.8^bcd^	16 ± 0.8^a^	13 ± 0.65^a^	14 ± 0.7^cde^	12 ± 0.6^de^		22 ± 1.1^ab^	19.5 ± 0.98^b^	18 ± 0.9^a^	16 ± 0.8^abcd^	15.5 ± 0.77^a^	12 ± 0.6^bc^	17 ± 0.85^bcd^	17 ± 0.85^bc^	12 ± 0.6^b^
5	14 ± 0.70^def^	14 ± 0.7^bcd^		17 ± 0.85^bc^	14 ± 0.7^abc^		15 ± 0.75^bcd^	12 ± 0.6^de^		14 ± 0.7^h^	14 ± 0.7^def^		16 ± 0.8^abcd^	16 ± 0.8^a^	12 ± 0.6^bc^	17 ± 0.85^bcd^	17 ± 0.85^bc^	12 ± 0.6^b^
6	15.5 ± 0.77^bcd^	15 ± 0.75^bc^		18 ± 0.9^ab^	16 ± 0.8^a^		14 ± 0.7^cde^			17.5 ± 0.88^defg^	17 ± 0.85^c^		15.5 ± 0.77^bcd^	15 ± 0.75^ab^	12 ± 0.6^bc^	23 ± 1.15^a^	22 ± 1.1^a^	15 ± 0.75^a^
7	16 ± 0.80^abcd^	14 ± 0.7^bcd^		17 ± 0.85^bc^	14 ± 0.7^abc^		15 ± 0.75^bcd^	14 ± 0.7^bcd^		15 ± 0.75^gh^	15 ± 0.75^cde^		16.5 ± 0.83^abc^	16 ± 0.8^a^	13 ± 0.65^a^			
8	12 ± 0.60^f^			12 ± 0.6^ef^			12 ± 0.6^ef^			15 ± 0.75^gh^	13 ± 0.65^ef^		15 ± 0.75^cde^	12 ± 0.6 cd				
9	13 ± 0.65^def^	12 ± 0.6^de^					17 ± 0.85^ab^	15 ± 0.75^abc^	11 ± 0.55^d^	14 ± 0.7^h^	12 ± 0.6^f^		16 ± 0.8^abcd^	13 ± 0.65^bcd^		14 ± 0.7^efg^	12 ± 0.6^f^	
10				15 ± 0.75 cd	12 ± 0.6^c^		18 ± 0.9^a^	17 ± 0.85^a^	12 ± 0.6 cd	17 ± 0.85^efg^	15 ± 0.75^cde^		12 ± 0.6^ef^	11 ± 0.55^d^		15 ± 0.75^def^	15 ± 0.75^cde^	
11	14 ± 0.70^def^	12 ± 0.6^de^		15 ± 0.75 cd			15 ± 0.75^bcd^	14 ± 0.7^bcd^		19 ± 0.95^cde^	16 ± 0.8 cd	12 ± 0.6^c^	15 ± 0.75^cde^	15 ± 0.75^ab^	12 ± 0.6^bc^	19 ± 0.95^b^	19 ± 0.95^b^	11 ± 0.55^b^
12													13 ± 0.65^ef^					
13				15.5 ± 0.77 cd	14 ± 0.7^abc^					15 ± 0.75^gh^	12 ± 0.6^f^		14 ± 0.7^def^	12 ± 0.6 cd		13 ± 0.65^fg^	13 ± 0.65^ef^	
14	18 ± 0.90^a^	18 ± 0.9^a^		15 ± 0.75 cd	13 ± 0.65^bc^		17 ± 0.85^ab^	17 ± 0.85^a^		20 ± 1^bcd^	13 ± 0.65^ef^		16 ± 0.8^abcd^	14 ± 0.7^abc^		16 ± 0.8^cde^	16 ± 0.8 cd	12 ± 0.6^b^
15	17.5 ± 0^.^88^ab^	16 ± 0.8^ab^		20 ± 1^a^	16 ± 0.8^a^		16 ± 0.8^abc^	16 ± 0.8^ab^	14 ± 0.7^ab^	20 ± 1^bcd^	16 ± 0.8 cd		18 ± 0.9^a^	15 ± 0.75^ab^	14 ± 0.7^a^	17 ± 0.85^bcd^	13.5 ± 0.65^ef^	12 ± 0.6^b^
16	12 ± 0.60^f^			16 ± 0.8^bcd^	15 ± 0.75^ab^		11 ± 0.55^f^	11 ± 0.55^e^		18 ± 0.9^def^	14 ± 0.7^def^		14 ± 0.7^def^	14 ± 0.7^abc^		16 ± 0.8^cde^	15 ± 0.75^cde^	
17	15 ± 0.75^cde^						14 ± 0.7^cde^			17 ± 0.85^efg^						14.5 ± 0.73^defg^	14 ± 0.7^def^	
18	15.5 ± 0.77^bcd^	15 ± 0.75^bc^		17 ± 0.85^bc^	15 ± 0.75^ab^		16 ± 0.8^abc^	13 ± 0.65^cde^	12 ± 0.6 cd	16 ± 0.8^fgh^	13 ± 0.65^ef^		14 ± 0.7^def^	12 ± 0.6 cd	11 ± 0.55^c^	15 ± 0.75^def^	14 ± 0.7^def^	
19				14 ± 0.7^de^	13 ± 0.65^bc^		14 ± 0.7^cde^	14 ± 0.7^bcd^		15 ± 0.75^gh^	12 ± 0.6^f^		13 ± 0.65^ef^					
20	13 ± 0.65^ef^			16 ± 0.8^bcd^	12 ± 0.6^c^								12 ± 0.6^ef^			18 ± 0.9^bc^	14 ± 0.7^def^	
21	15 ± 0.75^cde^	12 ± 0.6^de^		14 ± 0.7^de^	14 ± 0.7^abc^	12 ± 0.60^a^	17.5 ± 0.88^a^	17 ± 0.85^a^	15 ± 0.7^a^	17 ± 0.85^efg^			17 ± 0.85^abc^	14 ± 0.7^abc^	12 ± 0.6^bc^	15.5 ± 0.77^cdef^	15 ± 0.75^cde^	12 ± 0.6^b^
22	13 ± 0.65^ef^	12 ± 0.6^de^		12 ± 0.6^ef^	12 ± 0.6^c^		13 ± 0.65^def^	13 ± 0.65^cde^								12 ± 0.6^g^	12 ± 0.6^f^	
23	12 ± 0.6^f^												13 ± 0.65^ef^			12 ± 0.6^g^	12 ± 0.6^f^	
24	14 ± 0.7^def^	13 ± 0.65^cde^		15.5 ± 0.77 cd	12 ± 0.6^c^		15 ± 0.75^bcd^	15 ± 0.75^abc^		17.5 ± 0.88^defg^	17 ± 0.85^c^	15 ± 0.75^b^	16 ± 0.8^abcd^	13 ± 0.65^bcd^				
25	16 ± 0.8^abcd^	15 ± 0.75^bc^		15.5 ± 0.77 cd	15 ± 0.75^ab^	12 ± 0.60^a^	15 ± 0.75^bcd^	15 ± 0.75^abc^	13 ± 0.65^bc^	24 ± 1.2^a^	24 ± 1.2^a^	18 ± 0.9^a^	15.5 ± 0.77^bcd^	15 ± 0.75^ab^	12 ± 0.6^bc^	15.5 ± 0.77^cdef^	15 ± 0.75^cde^	12 ± 0.6^b^
26	16 ± 0.8^abcd^			16 ± 0.8^bcd^	15 ± 0.75^ab^		14 ± 0.7^cde^			18 ± 0.9^def^			17 ± 0.85^abc^	16 ± 0.8^a^	13 ± 0.65^a^	17 ± 0.85^bcd^	16 ± 0.8 cd	14 ± 0.7^a^
27	17 ± 0.85^abc^	15 ± 0.75^bc^		16 ± 0.8^bcd^	15.5 ± 0.77^a^	13 ± 0.65^a^	18 ± 0.9^a^	17 ± 0.85^a^	14 ± 0.7^ab^	21 ± 1.05^bc^	16 ± 0.8 cd		18 ± 0.9^a^	14 ± 0.7^abc^		18 ± 0.9^bc^	14 ± 0.7^def^	12 ± 0.6^b^
28				16 ± 0.8^bcd^	14 ± 0.7^abc^		16 ± 0.8^abc^	14 ± 0.7^bcd^		15 ± 0.75^gh^			16.5 ± 0.83^abc^	14 ± 0.7^abc^		16 ± 0.8^cde^	13 ± 0.65^ef^	
29				11 ± 0.55^f^									13 ± 0.65^ef^					
30																		

Different superscript letters (a, b, c...) within the same column and bacterial species indicate significant differences (p < 0.05) between honey concentrations according to Tukey’s post hoc test. Superscript letters are not comparable between different bacterial species.

Significant variations were observed among honey samples in their activity against *E. faecalis*, with inhibition zones ranging from 11 ± 0.55 mm to 24 ± 1.2 mm, making it the bacterium against which the strongest antimicrobial activity was recorded. The inhibition zones for *P. aeruginosa* ranged from a minimum of 12 ± 0.6 mm to a maximum of 19 ± 0.95 mm, while those for *B. subtilis* varied between 12 ± 0.6 mm and 18 ± 0.9 mm.

A general increase in inhibition zone diameters was observed with increasing honey concentrations, with the highest antimicrobial activity detected at a 75% concentration. However, certain honey samples exhibited weak or no activity against specific bacteria. When evaluated according to all concentrations, it was determined that samples showed antibacterial activity against Gram-negative bacteria, as in many studies, but a stronger antibacterial activity against Gram-positive bacteria [38–40]. This difference in antimicrobial activity can be attributed to the structural composition of Gram-positive bacteria, which have a thick peptidoglycan layer and lack an outer membrane, making them more susceptible to antibacterial compounds [41]. In contrast, Gram-negative bacteria possess an outer membrane containing lipopolysaccharides, which, along with their thin peptidoglycan layer, restricts the entry of antibacterial agents into the cell [42].

Statistically significant correlations were identified between the antimicrobial activity of honey samples and their chemical and physical parameters (Table 3). Diastase activity exhibited a strong positive correlation with *S.aureus* (r = 0.704, p < 0.01) and *E. faecalis* (r = 0.518, p < 0.01), whereas lower correlation values were observed for Gram-negative bacteria. Proline content was significantly correlated with *K. pneumoniae* (r = 0.454, p < 0.05), *P. aeruginosa* (r = 0.384, p < 0.05), *S. aureus* (r = 0.439, p < 0.05), and *B. subtilis* (r = 0.435, p < 0.05). Free acidity and electrical conductivity showed a notable positive correlation with the antibacterial activity of honey samples against all tested bacteria, with particularly strong correlations observed for *S. aureus* (r = 0.527, p < 0.01) and *E. faecalis* (r = 0.460, p < 0.05). The HMF content exhibited a negative correlation with *E. faecalis* (r = −0.364, p < 0.05), suggesting that high HMF levels may reduce antibacterial activity. Similarly, sucrose content displayed a negative correlation with the antibacterial activity against *E. faecalis* (r = −0.538, p < 0.01) and *E. coli* (r = −0.507, p < 0.01), indicating that honey samples with lower sucrose content exhibited higher antimicrobial activity. These findings, consistent with previous studies, highlight that the antibacterial efficacy of honey is directly related to its chemical composition, with honey samples exhibiting high diastase activity, free acidity, and electrical conductivity demonstrating greater antibacterial potency against bacteria [43,44]. In addition to HMF, several other compounds contribute to the antimicrobial activity of honey. These include hydrogen peroxide, generated via the action of glucose oxidase; bee-derived antimicrobial peptides such as defensin-1; and various phytochemicals of floral origin, particularly flavonoids (e.g., pinocembrin, chrysin, galangin), phenolic acids (e.g., caffeic acid), and alkaloids. These compounds have been shown to exert antimicrobial effects through mechanisms such as membrane disruption, inhibition of quorum sensing, and interference with efflux pumps and other resistance mechanisms [45,46].

**Table 3:** Calculated correlations. S: samples, D:diastase, P: proline, I:insoluble matter, M:moisture, F:free acidity, E:electrical conductivity, P:pH, H:HMF.

	S	D	P	I	M	F	E	P	H	Fructose	Glucose	Sucrose	Maltose	TPC-10 g	*E.coli*	*K.pneumoniae*	*S.aureus*	*E.faecalis*	*P.aeruginosa*	*B.subtilis*
S		−.159	−.127	−.320	−.016	−.022	−.201	−.558^**^	−.071	−.075	−.285	−.007	−.011	−.152	−.209	−.172	−.068	.069	.067	−.026
D			.667^**^	.378^*^	.533^**^	.754^**^	.746^**^	−.062	−.449^*^	.034	.442^*^	−.406^*^	−.506^**^	−.131	.645^**^	.469^**^	.704^**^	.518^**^	.547^**^	.421^*^
P				.255	.456^*^	.853^**^	.789^**^	−.355	−.119	−.143	.519^**^	−.146	−.551^**^	−.322	.335	.454^*^	.439^*^	.281	.384^*^	.435^*^
I					.257	.229	.210	−.095	−.149	.239	.312	−.147	−.275	−.090	.335	.206	.261	.111	.299	.260
M						.431^*^	.388^*^	−.248	−.202	−.243	.429^*^	−.411^*^	−.801^**^	−.144	.314	.096	.395^*^	.163	.188	.104
F							.904^**^	−.284	−.267	−.154	.511^**^	−.320	−.522^**^	−.494^**^	.482^**^	.372^*^	.527^**^	.460^*^	.476^**^	.407^*^
E								.026	−.122	−.102	.561^**^	−.352	−.466^**^	−.218	.458^*^	.433^*^	.513^**^	.406^*^	.379^*^	.336
P									.164	.072	−.025	−.074	.350	.448^*^	.044	−.063	−.121	.015	−.200	−.215
H										.066	.025	.135	.191	.281	−.250	−.132	−.098	−.364^*^	−.132	−.285
Fructose											.143	−.184	.249	.137	.143	.102	.152	.095	−.015	.027
Glucose												−.179	−.453^*^	−.153	.344	.224	.425^*^	−.046	.065	−.025
Sucrose													.453^*^	.134	−.507^**^	−.192	−.358	−.538^**^	−.310	−.077
Maltose														.242	−.231	−.139	−.246	−.133	−.180	.006
TPC-10 g															−.234	.004	−.097	−.168	−.164	−.199

**. Correlation is significant at the 0.01 level (2-tailed).

*. Correlation is significant at the 0.05 level (2-tailed).

### Insoluble matter content

The insoluble matter content in honey is a crucial indicator of its purity and processing quality. Insoluble solids, such as wax particles, pollen grains, and environmental contaminants, can affect the sensory properties and consumer acceptance of honey [47]. Inadequate filtration or improper handling can increase these levels, whereas effective purification techniques can significantly reduce them, thus improving the product’s cleanliness and marketability [48].

In this study, the insoluble matter content in Kars honey samples ranged from 0.02% to 0.08%, with an average of 0.03 ± 0.01% (Fig 1). These values are notably lower than those reported in honeys from Ethiopia (0.66–0.73%), Ghana (0.56–8.50%), and Algeria (0.01–0.67%) [49–51]. The low values observed may reflect effective processing and filtration practices in the region.

Several factors can influence insoluble matter levels, including botanical origin, pollen concentration, and environmental conditions. For instance, honeys with high pollen content often exhibit elevated insoluble matter values [52]. Likewise, samples produced in or near urban areas may show higher levels due to environmental pollutants [53]. However, despite originating from a mountainous region rich in meadow flora—conditions typically associated with higher pollen and wax content-the Kars honeys showed consistently low levels. This suggests that hygienic harvesting methods and traditional beekeeping practices may have helped limit contamination.

Overall, these findings indicate that Kars honey meets international purity standards and highlight the importance of good manufacturing practices to ensure both product quality and consumer safety.

### Diastase activity

Diastase activity is a significant parameter in determining honey quality. The diastase enzyme present in honey converts starch into simpler sugars, and its activity provides insight into the freshness and processing conditions of honey [4,54]. Diastase activity is influenced by various factors, including floral origin, climatic conditions, and storage duration. Exposure to heat and long-term storage can significantly reduce enzymatic activity [55]. Therefore, measuring diastase activity is critical for assessing honey freshness and detecting possible heat-related degradation.

According to the Codex Alimentarius and European Union standards, honey must have a minimum diastase number of 8 DN to be considered of acceptable quality [56]. Honey with lower values may indicate heat treatment, poor storage, or adulteration. In this study, the diastase activity of Kars honey samples ranged from 4.93 DN to 28.1 DN, with an average of 16.85 ± 6.61 DN (Fig 1). Although a few samples fell below the standard threshold, most samples demonstrated values above 8 DN, indicating acceptable quality.

These findings are comparable with previous studies from various countries. For instance, Czech honeys showed a range of 16–25.1 DN, Spanish honeys 10.1–44 DN, Estonian honeys 15.4–58.8 DN, and Brazilian honeys 15.5–41.4 DN [57–60]. Within Türkiye, diastase values reported in Bingöl honeys ranged from 17.89 to 28.93 DN, and in Ordu honeys from 15.80 to 39.30 DN [61,62]. Compared to these, Kars honey samples showed slightly lower minimum values but similar average levels.

The observed variation in diastase activity may be attributed to differences in botanical origin, harvest time, and beekeeping practices in the region. Moreover, the relatively lower values in some samples could result from mild thermal exposure or extended storage prior to analysis. Overall, the findings suggest that the majority of Kars honeys retain sufficient enzymatic activity and can be considered fresh and minimally processed.

### Electrical conductivity

Electrical conductivity is a key physicochemical parameter that provides insight into the mineral content, organic acids, and ionic composition of honey. It is also used to distinguish between blossom honeys (typically < 0.8 mS/cm) and honeydew honeys (typically > 0.8 mS/cm), as recommended by international standards [28,63]. Higher Electrical conductivity values are often associated with honeydew content, while lower values indicate floral origin.

In this study, the Electrical conductivity of Kars honey samples ranged from 0.138 to 0.343 mS/cm, with an average of 0.21 ± 0.05 mS/cm (Fig 1). These results indicate that all samples fall well within the range for blossom honey, which is consistent with the melissopalynological finding that most samples were multifloral.

The Electrical conductivity values observed in this study are comparable with those reported in several international and national studies. Honey samples from Kosovo (0.11–0.75 mS/cm) and Estonia (0.2–0.8 mS/cm) demonstrated similar ranges [59,64]. Likewise, honeys from Ordu, Türkiye, reported Electrical conductivity values between 0.20 and 0.21 mS/cm, closely matching the averages found in Kars [62]. In contrast, honeydew honeys from Greece and Brazil generally exhibit higher Electrical conductivity values due to their distinct botanical sources [60,65].

These findings support the classification of Kars honey as floral in origin and of high quality. The relatively narrow Electrical conductivity range also suggests consistent nectar sources and minimal contamination or adulteration. Since Electrical conductivity is affected by the floral spectrum and soil mineral composition, the values recorded in this study reflect the specific ecological conditions of the Kars region.

### Free acidity

Free acidity is an essential physicochemical parameter that reflects the quality, freshness, and stability of honey. It mainly arises from organic acids, especially gluconic acid, produced by the enzymatic oxidation of glucose during nectar processing by bees [4]. Higher acidity levels may indicate fermentation, poor storage, or botanical origin with naturally higher acid content [66].

In this study, the free acidity of Kars honey samples ranged from 13.9 to 31.5 meq/kg, with an average of 20.62 ± 5.06 meq/kg (Fig 1). All values were below the Codex Alimentarius maximum limit of 50 meq/kg, indicating acceptable freshness and absence of fermentation [56].

These findings are similar to those reported in other studies from Türkiye (e.g., 17.3–29.6 meq/kg in Bingöl and 21.5–34.1 meq/kg in Bayburt) and abroad (e.g., 17–30 meq/kg in Romanian and Estonian honeys) [59,61,63,67]. Such similarity suggests a comparable floral origin and harvesting process in these regions.

Additionally, free acidity showed positive correlations with other quality parameters such as diastase activity, proline, and Electrical conductivity, supporting its role as an indicator of honey maturity and biochemical complexity.

### HMF

HMF is a thermal degradation product of sugars and serves as a key indicator of honey freshness and potential heat treatment. Its concentration increases with prolonged storage, high processing temperatures, or adulteration with inverted sugars [68]. International regulations, including those of the Codex Alimentarius, set a maximum limit of 40 mg/kg for most honeys and 80 mg/kg for tropical varieties [69].

**Fig 2:**
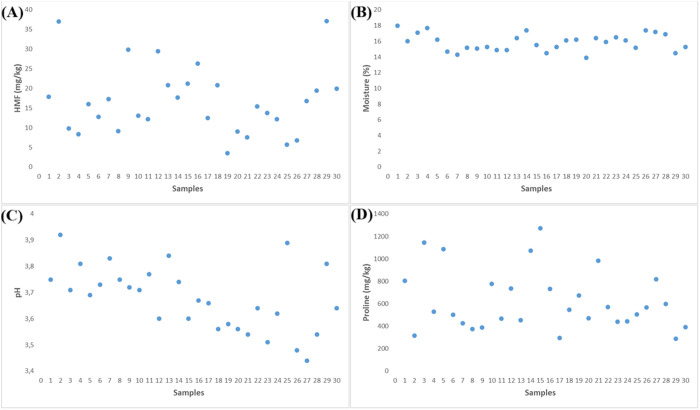
Additional quality parameters of honey samples: (A) HMF (mg/kg), (B) Moisture (%), (C) pH, and (D) Proline (mg/kg).

In the present study, the HMF content of Kars honey samples ranged from 3.5 to 37.1 mg/kg, with an average of 16.65 ± 8.59 mg/kg (Fig 2). These values fall well within acceptable limits, indicating that the samples were properly stored and had not undergone excessive heating.

Comparable HMF levels have been reported in honeys from Romania (6.5–19.1 mg/kg), Estonia (3.5–19.5 mg/kg), and Bingöl, Türkiye (7.1–11.9 mg/kg) [59,61,70]. In contrast, higher HMF values were detected in honey from Morocco (18.6–46.5 mg/kg) and the Western Balkans, likely due to climatic and processing differences [71,72].

While HMF levels in this study do not indicate any quality defects, it is important to note that HMF has been reported to exhibit cytotoxic and mutagenic effects at high concentrations, reinforcing the need to minimize its presence in honey intended for human consumption [68]. The low HMF values observed in Kars honey suggest that it was harvested and stored under suitable conditions, preserving both its quality and safety.

### Moisture content

Moisture content is a critical quality parameter in honey, directly influencing its stability, shelf life, and risk of fermentation. According to the Codex Alimentarius, honey should contain less than 20% moisture, with values between 14% and 18% considered optimal [73]. Higher moisture levels may promote fermentation and microbial growth, while lower values contribute to extended preservation.

In this study, the moisture content of Kars honey samples ranged from 13.9% to 18.0%, with an average of 15.87 ± 1.08% (Fig 2). These values are well within the acceptable range and indicate that the honey was properly matured and stored.

Similar results have been reported for Turkish honeys from Bayburt (14.8–18.4%) and Bingöl (15.0–17.2%), as well as for Estonian (15.6–20.9%) and Romanian honeys (15.2–17.3%) [59,61,67,70]. The relatively narrow moisture range observed in Kars honeys suggests standardized harvesting and post-harvest practices.

Overall, the moisture levels in Kars honeys reflect high quality and compliance with international standards. These values also support the absence of fermentation or adulteration and contribute positively to the sensory and physicochemical stability of the product.

### pH

The pH of honey is a key indicator of its freshness, microbial stability, and chemical composition. Typically ranging between 3.2 and 4.5, this acidic environment inhibits microbial growth and contributes to honey’s long shelf life [74]. Honey acidity primarily results from organic acids such as gluconic acid, produced during nectar processing by bees [4].

In this study, the pH of Kars honey samples ranged from 3.44 to 3.92, with an average of 3.68 ± 0.12 (Fig 2). These values are consistent with those reported in previous studies of Turkish and international honeys, including Bayburt (3.45–3.84), Romania (3.5–4.1), and Estonia (3.4–4.0) [59,67,70].

The narrow pH range suggests a stable chemical profile and appropriate processing conditions for Kars honey. Moreover, the acidic nature of the samples supports the absence of microbial fermentation and aligns with quality standards established by international guidelines.

### Proline

Proline is the most abundant amino acid in honey and serves as a marker of its maturity, botanical origin, and potential adulteration. Low proline levels (< 183 mg/kg) may suggest early harvest or sugar feeding, while high levels indicate natural origin and full ripening [75,76].

In this study, proline content in Kars honey samples ranged from 288.87 to 1272.9 mg/kg, with an average of 622.56 ± 267.66 mg/kg (Fig 2). These values are significantly above the international threshold and confirm the natural and mature status of the honey.

Comparable proline levels have been reported in honeys from Bingöl (438–965 mg/kg), Ordu (609–796 mg/kg), and Kayseri (981–1050 mg/kg), while Burdur honeys showed even higher values (1258–1404 mg/kg) [61,62,77]. Internationally, Estonian honeys showed a wide range (257–1328 mg/kg), and Nigerian honeys reported values near the lower limit [59,78].

Additionally, proline content has been positively correlated with antimicrobial activity, as confirmed in this study, where higher proline levels were associated with increased inhibition against several bacterial strains. This supports previous findings linking proline to the biological efficacy of honey [79].

### Sugar composition

The sugar composition of honey is a key indicator of its botanical origin, sweetness, crystallization tendency, and potential adulteration. Fructose and glucose are the major sugars, while sucrose and maltose are found in smaller amounts. The fructose/glucose ratio also affects honey’s physical properties, such as its tendency to remain liquid or crystallize [80,81].

**Fig 3:**
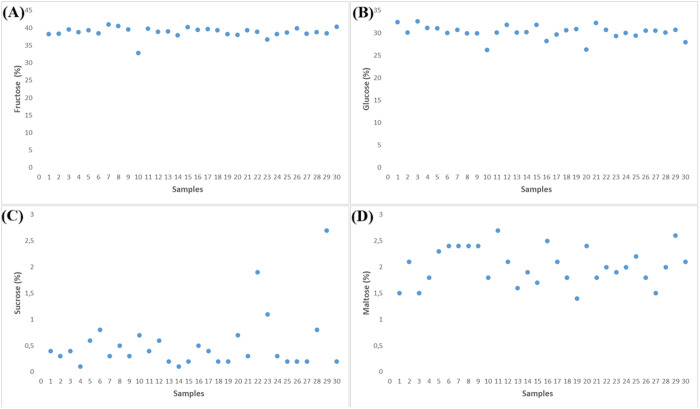
Sugar composition of honey samples: (A) Fructose (%), (B) Glucose (%), (C) Sucrose (%), and (D) Maltose (%).

In this study, the fructose content of Kars honey ranged from 32.8% to 41.0%, glucose from 26.2% to 32.6%, sucrose from 0.1% to 2.7%, and maltose from 1.4% to 2.7% (Fig 3). These values are within international quality standards and suggest natural, unadulterated honey.

Comparable sugar profiles were observed in honeys from Romania (fructose: 33.7–39.0%, glucose: 24.4–36.1%), Bayburt (fructose: 40.1–42.6%, glucose: 32.9–36.7%), and the Czech Republic (sucrose: 0.1–0.8%, maltose: 2.9–4.6%) [67,70,82]. The slightly lower sucrose levels in Kars samples further support the notion of proper harvesting and minimal manipulation.

The dominance of fructose over glucose indicates that Kars honey is less prone to crystallization, contributing to longer shelf life and consumer preference. In addition, the low sucrose values confirm the absence of added sugars and align with Codex Alimentarius standards [83]. Overall, the sugar composition supports the authenticity and high quality of Kars honey.

Overall, the findings of this study highlight that Kars honey exhibits a strong physicochemical and biological profile that aligns with international quality standards. The moisture, pH, HMF, diastase activity, and sugar content values confirm the freshness and authenticity of the samples, while melissopalynological analysis revealed a diverse floral origin, dominated by Fabaceae, Lamiaceae, and Boraginaceae. Furthermore, the antimicrobial activity, particularly against Gram-positive bacteria, supports the therapeutic potential of Kars honey. These results collectively demonstrate that the honey from this region is a high-quality, natural product with both nutritional and functional value.

## Conclusion

This study presents a comprehensive evaluation of honey samples from Kars Province, Türkiye, focusing on their physicochemical characteristics, pollen composition, and antimicrobial activity. The results confirmed that Kars honey meets international quality standards and contains a diverse floral spectrum dominated by Fabaceae, Lamiaceae, and Boraginaceae. Most samples were multifloral with high pollen counts, reflecting the ecological richness of the region.

The honey samples showed notable antimicrobial activity, particularly against Gram-positive bacteria. Positive correlations between antibacterial effects and parameters such as proline content, diastase activity, and free acidity suggest synergistic biochemical contributions.

However, the study has some limitations. Antimicrobial activity was assessed using only the agar well diffusion method, and sampling was restricted to a single region and season. Although this method allowed for comparative screening and is widely used in honey research, we acknowledge that more quantitative methods (e.g., MIC/MBC assays) would have provided greater precision. Future studies will incorporate more advanced antimicrobial testing protocols. Additionally, parameters such as antioxidant capacity, mineral content, and enzyme activity were not examined. Future research should include broader sampling and expanded biochemical analyses to better understand the therapeutic potential of honey.

Overall, the findings support the recognition of Kars honey as a high-quality natural product with both nutritional and medicinal value.

## Supporting information

S1 Table Insoluble matter content, diastase activity, electrical conductivity, free acidity, hydroxymethylfurfural, moisture content, pH, proline, sugar profile and TPC-10 g values. (DOCX)
