# Supplementary material for: Multidimensional analysis of honey from Eastern Anatolia (Kars): Pollen spectrum, physicochemical properties, and antimicrobial activity
Source: PLoS One. 2025 Jul 9;20(7):e0327861. doi: 10.1371/journal.pone.0327861 (PMC12240352; doi:10.1371/journal.pone.0327861)
Supplement: S1 Table — (DOCX) [file pone.0327861.s001.docx]

**S1 Table.** **Insoluble matter content, diastase activity, electrical conductivity, free acidity, hydroxymethylfurfural, moisture content, pH, proline, sugar profile and TPC-10 values**

| **Samples** | **Diastase activity** | **Proline** | **Insoluble**  **matter** | **Moisture** | **Free acidity** | **EC** | **pH** | **HMF** | **Fructose** | **Glucose** | **Sucrose** | **Maltose** | **TPC-10** |
| --- | --- | --- | --- | --- | --- | --- | --- | --- | --- | --- | --- | --- | --- |
| **1** | 28.10±4.02^a^ | 805.46±118.32^bcd^ | 0.06±0.04^a^ | 18.0±0.3^a^ | 24.3±1.1^bcdef^ | 0.308±0.014^b^ | 3.75±0.17^a^ | 17.9±1.3^cdefg^ | 38.3±1.0^ab^ | 32.4±0.9^ab^ | 0.4±0.1^fgh^ | 1.5±0.2^fg^ | 48167±1204^gh^ |
| **2** | 4.93±0.70^k^ | 316.72±46.53^h^ | 0.03±0.03^a^ | 16.0±0.3^fghijk^ | 13.9±0.6^h^ | 0.143±0.006^lm^ | 3.92±0.17^a^ | 37.0±2.6^a^ | 38.4±1.0^ab^ | 30.1±0.8^abcdef^ | 0.3±0.1^ghi^ | 2.1±0.2^abcdefg^ | 32915±823^jkl^ |
| **3** | 27.4±3.92^ab^ | 1144.57±168.14^a^ | 0.03±0.02^a^ | 17.1±0.3^abcde^ | 24.4±1.1^bcdef^ | 0.288±0.013^bc^ | 3.71±0.16^a^ | 9.8±0.7^klmn^ | 39.6±1.0^ab^ | 32.6±0.9^a^ | 0.4±0.1^fgh^ | 1.5±0.2^fg^ | 67019±1675^f^ |
| **4** | 20.73±2.97^abcde^ | 528.05±77.57^defgh^ | 0.05±0.04^a^ | 17.7±0.3^ab^ | 24.8±1.1^bcde^ | 0.278±0.012^bcd^ | 3.81±0.17^a^ | 8.3±0.6^mn^ | 38.8±1.0^ab^ | 31.1±0.9^abcd^ | 0.1±0.1^i^ | 1.8±0.2^cdefg^ | 19328±483^pqr^ |
| **5** | 19.88±2.84^abcde^ | 1087.78±159.80^ab^ | 0.06±0.05^a^ | 16.2±0.3^efghi^ | 23.3±1.0^cdef^ | 0.250±0.011^def^ | 3.69±0.16^a^ | 16.0±1.1^efghij^ | 39.3±1.0^ab^ | 31.0±0.8^abcd^ | 0.6±0.1^def^ | 2.3±0.2^abcde^ | 44604±1115^hi^ |
| **6** | 15.44±2.21^defghi^ | 502.94±73.8^defgh^ | 0.03±0.02^a^ | 14.7±0.3^lmno^ | 17.3±0.8^g^ | 0.201±0.009^gh^ | 3.73±0.16^a^ | 12.8±0.9^ijkl^ | 38.5±1.0^ab^ | 30.0±0.8^abcdef^ | 0.8±0.1^d^ | 2.4±0.2^abcd^ | 101934±2548^d^ |
| **7** | 16.89±2.42^defgh^ | 424.85±62.41^fgh^ | 0.07±0.05^a^ | 14.3±0.3^no^ | 17.2±0.8^g^ | 0.189±0.008^hij^ | 3.83±0.17^a^ | 17.3±1.2^cdefgh^ | 41.0±1.1^a^ | 30.7±0.8^abcde^ | 0.3±0.1^ghi^ | 2.4±0.3^abcd^ | 68031±1701^f^ |
| **8** | 19.00±2.72^cdefg^ | 375.15±4.91^gh^ | 0.02±0.01^a^ | 15.2±0.3^jklmn^ | 15.6±0.7^gh^ | 0.143±0.006^lm^ | 3.75±0.17^a^ | 9.1±0.6^lmn^ | 40.6±1.1^a^ | 29.9±0.8^bcdef^ | 0.5±0.1^efg^ | 2.4±0.3^abcd^ | 45325±1133^gh^ |
| **9** | 11.25±1.60^ghijk^ | 388.26±57.04^gh^ | 0.03±0.02^a^ | 15.1±0.3^klmn^ | 16.1±0.7^gh^ | 0.162±0.007^jklm^ | 3.72±0.16^a^ | 29.9±2.1^b^ | 39.6±1.0^ab^ | 29.9±0.8^bcdef^ | 0.3±0.1^ghi^ | 2.4±0.3^abcd^ | 33739±843^jk^ |
| **10** | 19.54±2.80^bcdef^ | 775.43±113.9^bcde^ | 0.06±0.04^a^ | 15.3±0.3^ijklm^ | 23.3±1.0^cdef^ | 0.240±0.011^ef^ | 3.71±0.16^a^ | 13.1±0.9^hijkl^ | 32.8±0.8^c^ | 26.2±0.7^g^ | 0.7±0.1^de^ | 1.8±0.3^cdefg^ | 38789±970^ij^ |
| **11** | 19.51±2.79^bcdef^ | 466.35±68.51^efgh^ | 0.05±0.04^a^ | 14.9±0.3^lmn^ | 17.3±0.8^g^ | 0.196±0.009^hi^ | 3.77±0.17^a^ | 12.2±0.9^jklm^ | 39.8±1.0^ab^ | 30.1±0.8^abcdef^ | 0.4±0.1^fgh^ | 2.7±0.2^a^ | 104357±2609^d^ |
| **12** | 8.27±1.18^ijk^ | 736.66±108.21^cdef^ | 0.03±0.02^a^ | 14.9±0.3^lmn^ | 26.1±1.2^bc^ | 0.255±0.011^def^ | 3.60±0.16^a^ | 29.5±2.1^b^ | 38.9±1.0^ab^ | 31.8±0.9^abcd^ | 0.6±0.1^def^ | 2.1±0.2^abcdefg^ | 15181±380^rs^ |
| **13** | 11.46±1.64^fghijk^ | 452.7±66.5^fgh^ | 0.03±0.02^a^ | 16.4±0.3^defgh^ | 15.0±0.7^gh^ | 0.170±0.008^ijkl^ | 3.84±0.17^a^ | 20.8±1.5^cd^ | 39.0±1.0^ab^ | 30.1±0.8^abcdef^ | 0.2±0.1^hi^ | 1.6±0.2^efg^ | 278320±6958^a^ |
| **14** | 26.09±3.73^abc^ | 1072.49±157.5^ab^ | 0.03±0.02^a^ | 17.4±0.3^abc^ | 25.3±1.1^bcd^ | 0.273±0.012^cd^ | 3.74±0.16^a^ | 17.7±1.3^cdefg^ | 37.9±1.0^ab^ | 30.2±0.8^abcdef^ | 0.1±0.1^i^ | 1.9±0.2^bcdefg^ | 51317±1283^g^ |
| **15** | 27.46±3.93^ab^ | 1272.9±186.9^a^ | 0.06±0.05^a^ | 15.5±0.3^hijkl^ | 31.5±1.4^a^ | 0.286±0.013^bc^ | 3.60±0.16^a^ | 21.2±1.5^c^ | 40.2±1.0^a^ | 31.8±0.9^abcd^ | 0.2±0.1^hi^ | 1.7±0.2^defg^ | 26212±655^mno^ |
| **16** | 8.01±1.14^ijk^ | 732.83±107.6^cdef^ | 0.02±0.02^a^ | 14.5±0.3^mno^ | 17.2±0.8^g^ | 0.183±0.008^hijk^ | 3.67±0.16^a^ | 26.3±1.9^b^ | 39.5±1.0^ab^ | 28.2±0.8^efg^ | 0.5±0.1^efg^ | 2.5±0.2^abc^ | 30691±767^klm^ |
| **17** | 11.09±1.58^ghijk^ | 294.33±43.24^h^ | 0.08±0.06^a^ | 15.3±0.3^ijklm^ | 14.5±0.6^gh^ | 0.138±0.006^m^ | 3.66±0.16^a^ | 12.5±0.9^jklm^ | 39.7±1.0^ab^ | 29.7±0.8^cdef^ | 0.4±0.1^fgh^ | 2.1±0.3^abcdefg^ | 36977±924^j^ |
| **18** | 14.55±2.08^defghi^ | 546.62±80.3^defgh^ | 0.03±0.02^a^ | 16.1±0.3^fghij^ | 21.6±1.0^f^ | 0.206±0.009^gh^ | 3.56±0.16^a^ | 20.8±1.5^cd^ | 39.4±1.0^ab^ | 30.6±0.8^abcde^ | 0.2±0.1^hi^ | 1.8±0.2^cdefg^ | 26303±658^mno^ |
| **19** | 15.50±2.20^efghi^ | 673.31±98.91^cdefg^ | 0.03±0.02^a^ | 16.2±0.3^efghi^ | 23.9±1.1^bcdef^ | 0.230±0.010^fg^ | 3.58±0.16^a^ | 3.5±0.3^o^ | 38.3±1.0^ab^ | 30.9±0.8^abcd^ | 0.2±0.1^hi^ | 1.4±0.2^g^ | 27016±675^lmn^ |
| **20** | 13.32±1.91^efghij^ | 470.72±69.15^efgh^ | 0.03±0.02^a^ | 13.9±0.3^no^ | 16.7±0.7^gh^ | 0.145±0.006^lm^ | 3.56±0.16^a^ | 9.0±0.6^lmn^ | 38.0±1.0^ab^ | 26.3±0.7^g^ | 0.7±0.1^de^ | 2.4±0.1^abcd^ | 20803±520^opqr^ |
| **21** | 22.11±3.10^abcd^ | 982.39±144.31^abc^ | 0.04±0.03^a^ | 16.4±0.3^defgh^ | 25.0±1.1^bcde^ | 0.249±0.011^def^ | 3.54±0.16^a^ | 7.6±0.5n^o^ | 39.4±1.0^ab^ | 32.2±0.9^abc^ | 0.3±0.1^ghi^ | 1.8±0.3^cdefg^ | 9981±250^s^ |
| **22** | 14.28±2.00^defghi^ | 570.65±83.83^defgh^ | 0.03±0.02^a^ | 15.9±0.3^ghijk^ | 17.3±0.8^g^ | 0.165±0.007^jklm^ | 3.64±0.16^a^ | 15.4±1.1^fghij^ | 38.9±1.0^ab^ | 30.7±0.8^abcde^ | 1.9±0.2^b^ | 2.0±0.2^abcdefg^ | 24131±603^nopq^ |
| **23** | 10.09±1.44^hijk^ | 438.5±64.42^fgh^ | 0.03±0.02^a^ | 16.5±0.3^cdefg^ | 17.2±0.8^g^ | 0.153±0.007^klm^ | 3.51±0.15^a^ | 13.8±1^ghijk^ | 36.7±1.0^b^ | 29.3±0.8^def^ | 1.1±0.1^c^ | 1.9±0.2^bcdefg^ | 24515±613^mnop^ |
| **24** | 15.15±2.10^defghi^ | 445.05±65.38^fgh^ | 0.02±0.01^a^ | 16.1±0.3^fghij^ | 16.7±0.7^gh^ | 0.161±0.007^jklm^ | 3.62±0.16^a^ | 12.2±0.9^jklm^ | 38.3±1.0^ab^ | 30.0±0.8^abcdef^ | 0.3±0.1^ghi^ | 2.0±0.2^abcdefg^ | 83998±2100^e^ |
| **25** | 21.70±3.10^abcd^ | 506.21±74.36^defgh^ | 0.02±0.02^a^ | 15.2±0.3^jklmn^ | 26.6±1.2^b^ | 0.343±0.015^a^ | 3.89±0.17^a^ | 5.7±0.4^no^ | 38.7±1.0^ab^ | 29.4±0.8^def^ | 0.2±0.1^hi^ | 2.2±0.2^abcdef^ | 18132±453^qr^ |
| **26** | 20.19±2.80^abcde^ | 566.83±83.27^defgh^ | 0.08±0.06^a^ | 17.4±0.3^abc^ | 22.2±1.0^ef^ | 0.180±0.008^hijk^ | 3.48±0.15^a^ | 6.8±0.5^no^ | 39.9±1.0^ab^ | 30.5±0.8^abcdef^ | 0.2±0.1^hi^ | 1.8±0.2^cdefg^ | 22921±573^nopq^ |
| **27** | 27.70±3.90^ab^ | 818.02±120.17^bcd^ | 0.03±0.03^a^ | 17.2±0.3^abcd^ | 31.5±1.4^a^ | 0.263±0.012^cde^ | 3.44±0.15^a^ | 16.8±1.2^defghi^ | 38.4±1.0^ab^ | 30.5±0.8^abcdef^ | 0.2±0.1^hi^ | 1.5±0.2^fg^ | 19841±496^pqr^ |
| **28** | 16.66±2.30^defgh^ | 599.04±88^defgh^ | 0.03±0.02^a^ | 16.9±0.3^bcdef^ | 22.7±1.0^def^ | 0.197±0.009^hi^ | 3.54±0.16^a^ | 19.5±1.4^cdef^ | 38.8±1.0^ab^ | 30.1±0.8^abcdef^ | 0.8±0.1^d^ | 2.0±0.2^abcdefg^ | 50234±1256^gh^ |
| **29** | 5.13±0.70^jk^ | 288.87±42.44^h^ | 0.02±0.01^a^ | 14.5±0.3^mno^ | 14.0±0.6^h^ | 0.167±0.007^jklm^ | 3.81±0.17^a^ | 37.1±2.7^a^ | 38.5±1.0^ab^ | 30.7±0.8^abcde^ | 2.7±0.3^a^ | 2.6±0.3^ab^ | 131675±3292^c^ |
| **30** | 14.12±2.02^defghi^ | 393.17±57.76^gh^ | 0.03±0.02^a^ | 15.3±0.3^ijklm^ | 16.2±0.7^gh^ | 0.178±0.008^hijk^ | 3.64±0.16^a^ | 19.9±1.4^cde^ | 40.3±1.0^a^ | 27.9±0.8^fg^ | 0.2±0.1^hi^ | 2.1±0.2^abcdefg^ | 152317±3808^b^ |
